# Supplementary material for: Oncogenic PIK3CA mutations increase dependency on the mRNA cap methyltransferase, RNMT, in breast cancer cells
Source: Open Biol. 2019 Apr 17;9(4):190052. doi: 10.1098/rsob.190052 (PMC6501644; doi:10.1098/rsob.190052)
Supplement: Supplemental Figures S1 - S8 [file rsob190052supp1.pdf]

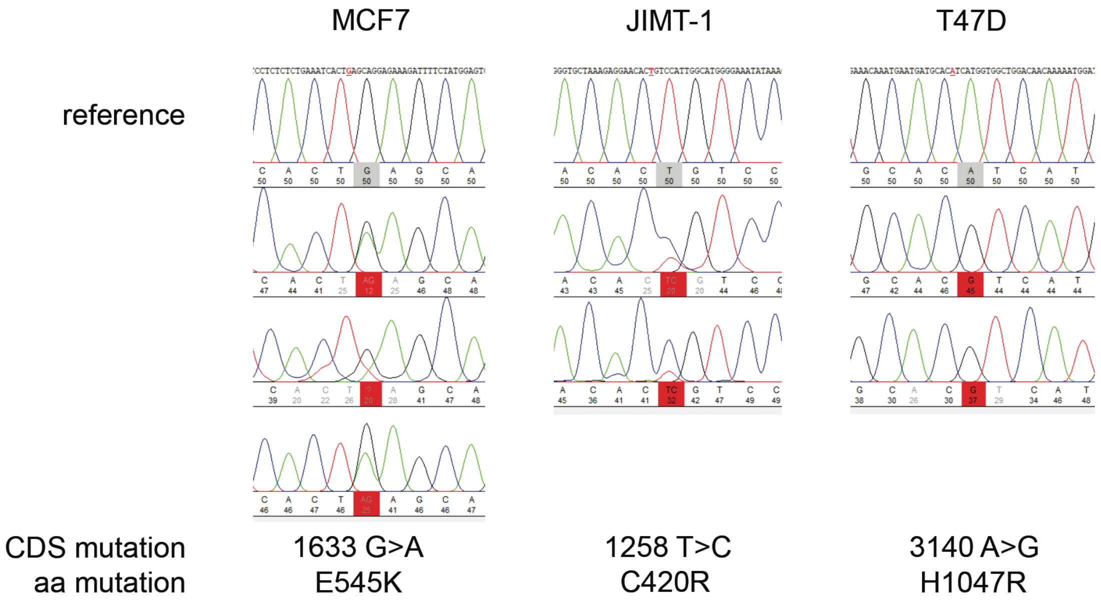

cell line	PIK3CA
MCF7	E545K
HCC1806	WT
JIMT-1	C420R
T47D	H1047R
CAMA-1	WT
BT-549	WT
MDA-MB-231	WT
ZR-75-1	WT

Supplemental Figure 1 Cell line PIK3CA coding sequence verification

The coding sequence of PIK3CA was verified for breast cancer cell lines. For MCF7, JIMT-1 and T47D, reported PIK3CA mutations were confirmed and no other mutations were detected. For HCC1806, CAMA-1, BT-549, MDA-MB-231 and ZR-75-1, PIK3CA was confirmed to be WT.

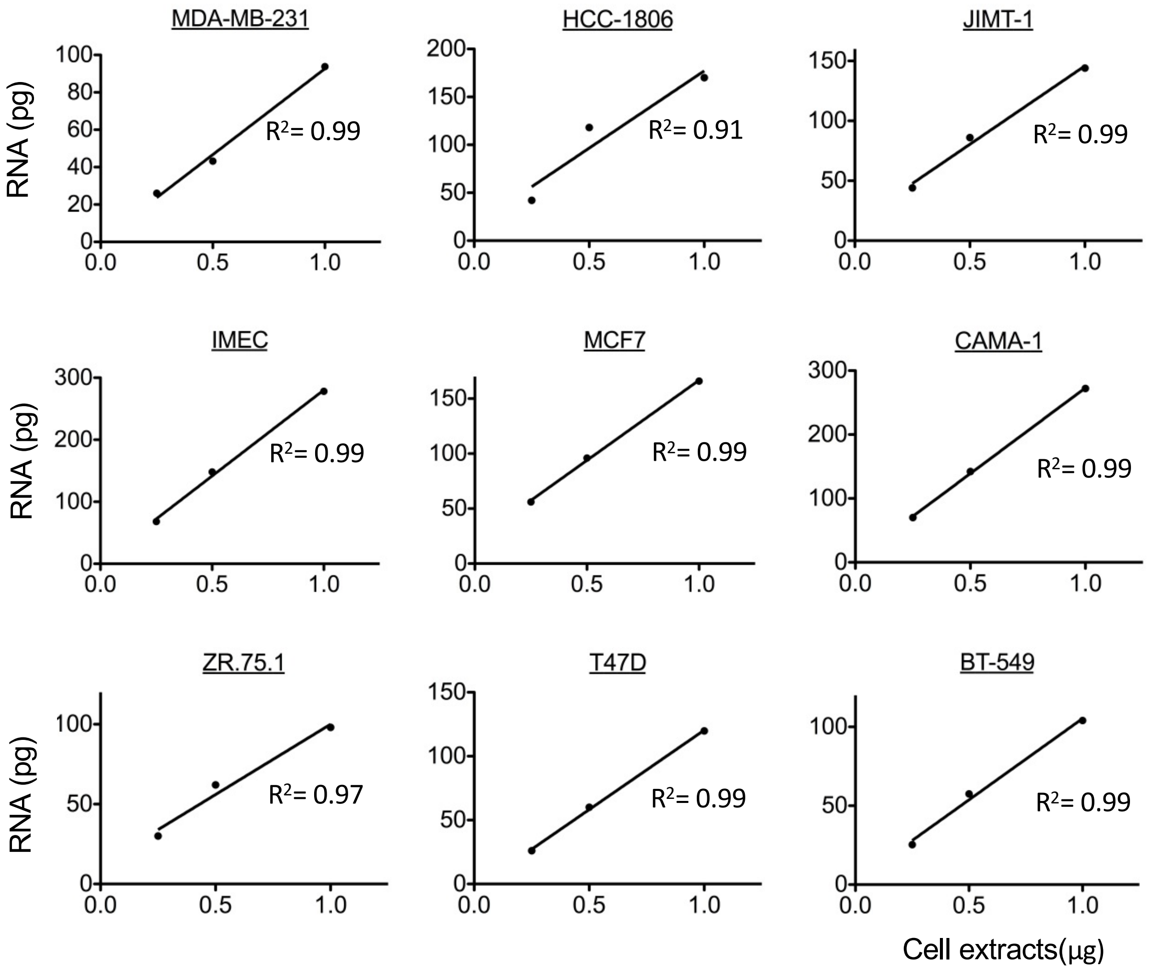

Supplemental Figure 2 mRNA cap methyltransferase assay

The *in vitro* N7 cap guanosine methyltransferase assay was performed using 0.25, 0.5 and 1 µg of cell extracts, or without extract as a negative control. The quantity of GpppG transcripts converted to m7GpppG transcripts in 10 minutes was determined. Each graph depicts the linear regression for the correlation of protein extract (µg) and the mean conversion of GpppG to m7GpppG. The linear regression coefficient (R^2) was determined using GraphPad Prism 5.0.

Supplemental Figure 3

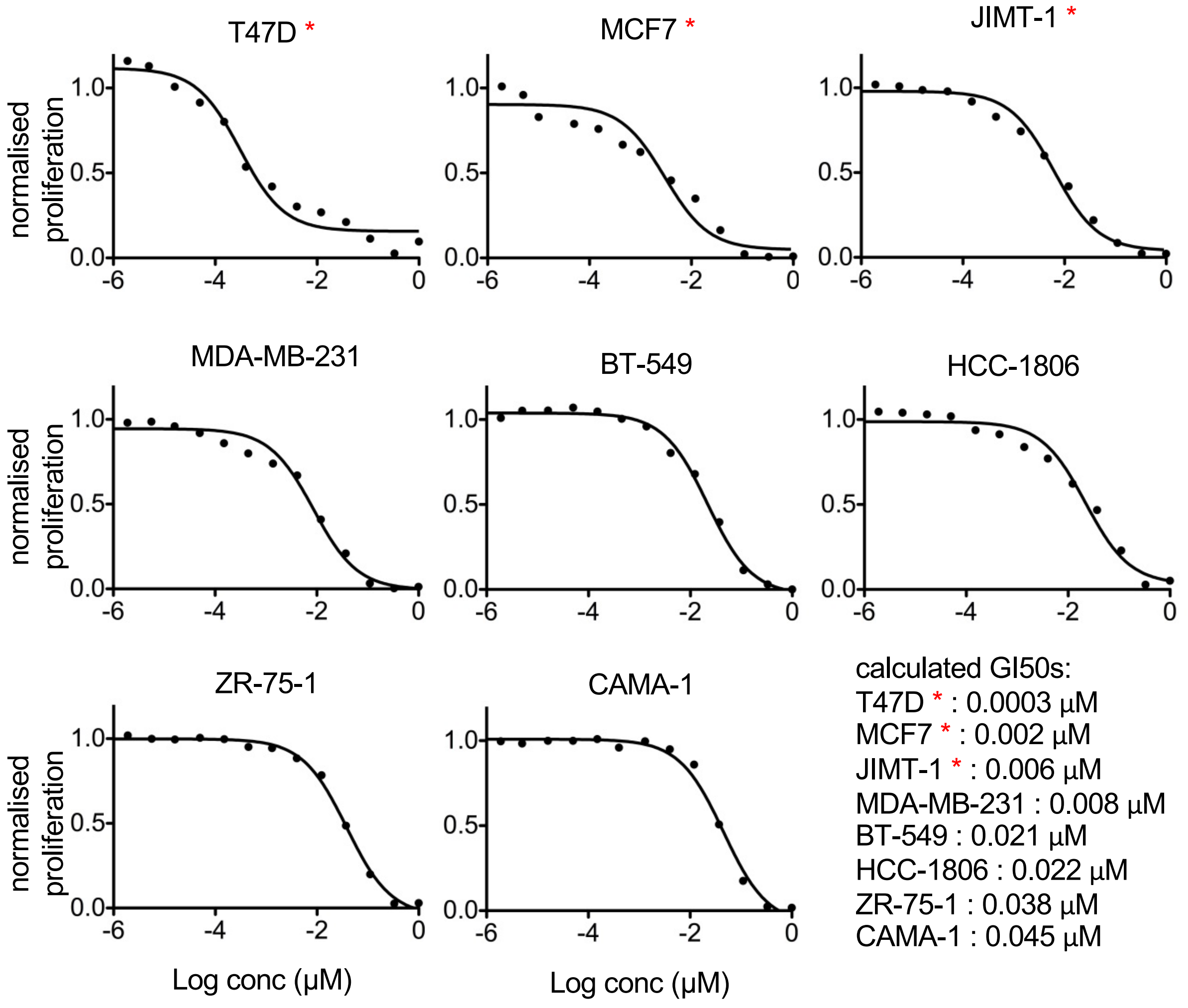

Supplemental Figure 3 Dose response curve for cell lines treated with PIK3CA inhibitor, BYL719

Cell lines were incubated with a titration of BLY719 from 1 μM-0.001 nM for 72 hours. Viable cells were determined using Cell-Titer Blue assay (Promega). The data fitted to dose response curve and GI50 values were calculated using GraphPad Prism 5.0.

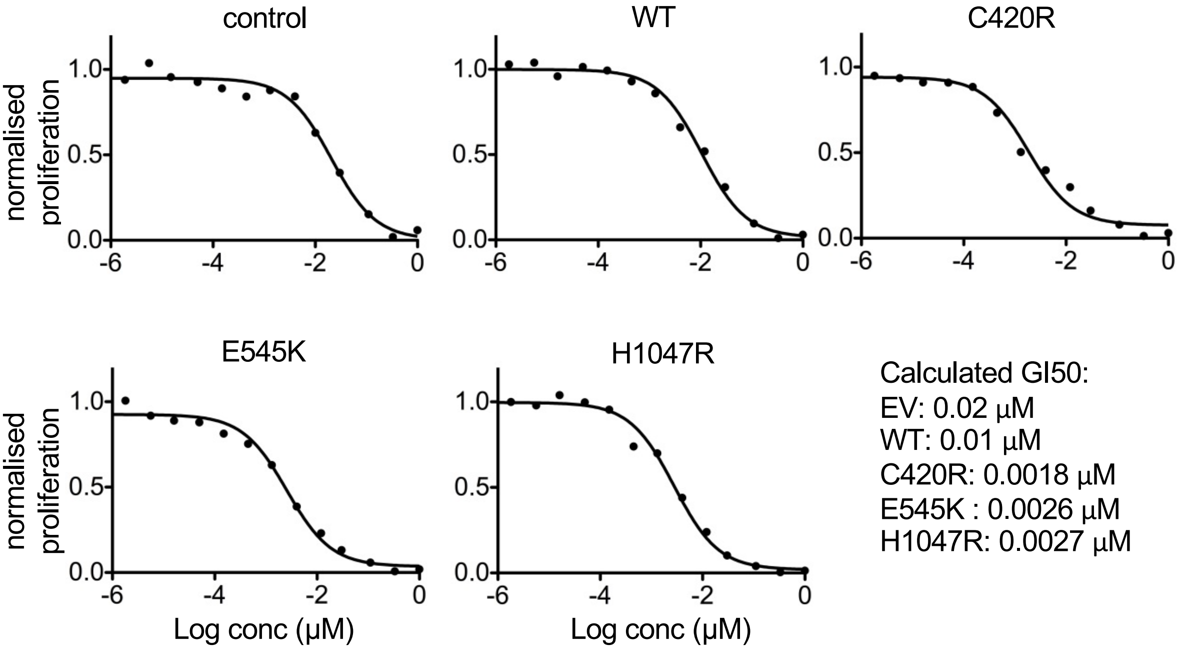

Supplemental Figure 4 Dose response curves of ZR-75-1 cell lines expressing PI3K p110α WT and mutants treated with p110α inhibitor, BYL719.

Cells expressing PIK3CA WT, C420R, E545K, H1047R or vector control were incubated in a titration of BYL719 from 1 μM-0.001 nM for 72 hours. Viable cells were determined using Cell-Titer Blue assay (Promega). Data were fitted to a dose response curve and GI50 values calculated using GraphPad Prism 5.0.

Supplemental Figure 5

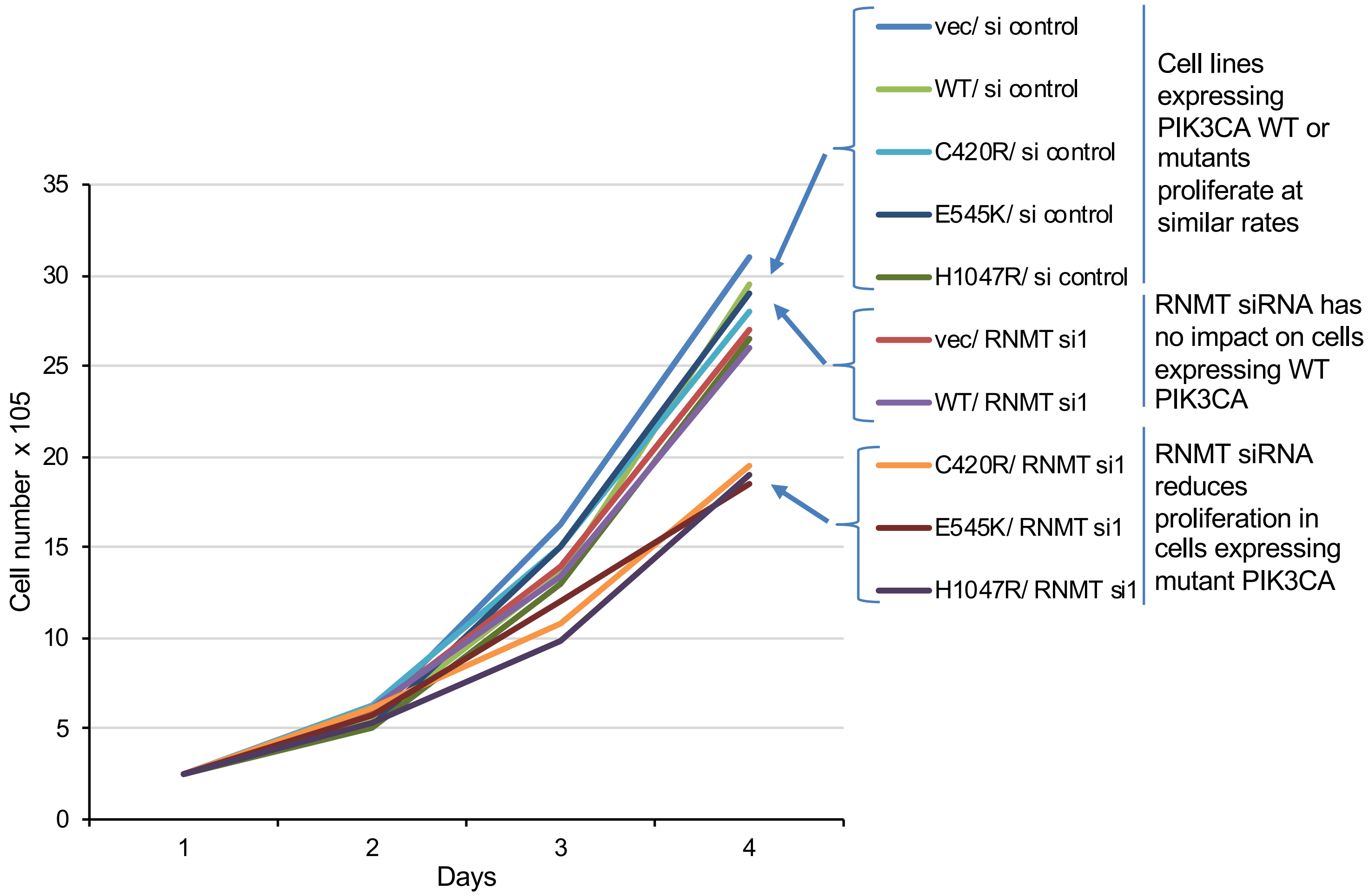

Supplemental Figure 5 Analysis of cell number following RNMT siRNA transfection of ZR-75-1/ PI3K p110α cell lines

ZR-75-1/ PI3K p110α cell lines were transfected with RNMT siRNA1 or non-targeting control for 48hrs. Cell number was determined over 3 days. Average cell number from three independent transfections is reported. The data is the same as presented in figure 4d.

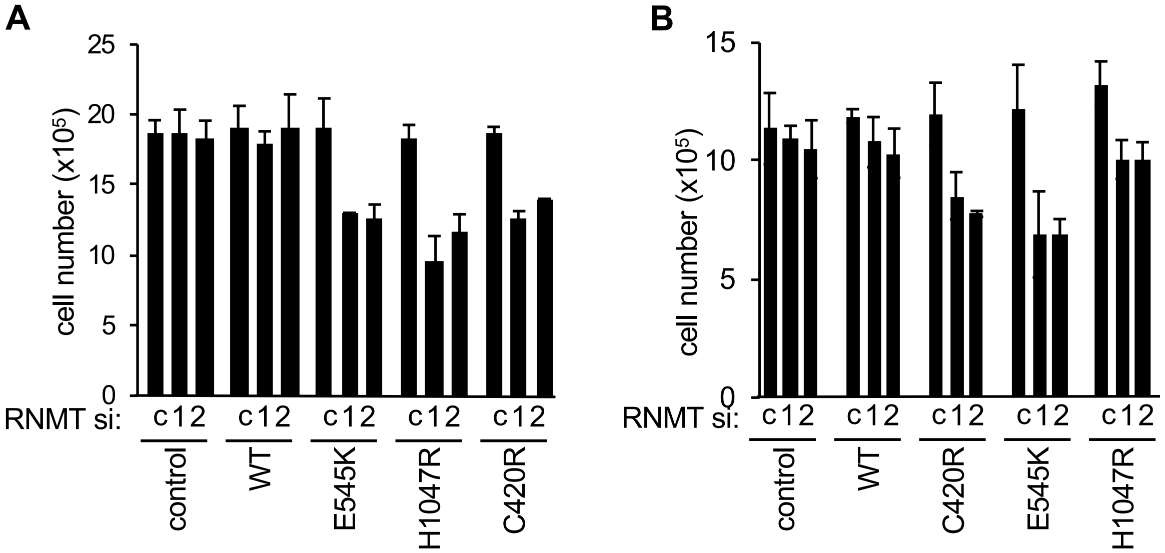

Supplemental Figure 6 Analysis of cell number following two independent RNMT siRNAs

Cell lines expressing PIK3CA WT, mutants or controls were plated as follows a) 2.5 x10^5 IMEC, and b) 1.5 x 10^5 MDA-MB-231. Cells were transfected with two independent RNMT siRNAs or control. After 72hrs cells were counted. Average of three independent experiments presented

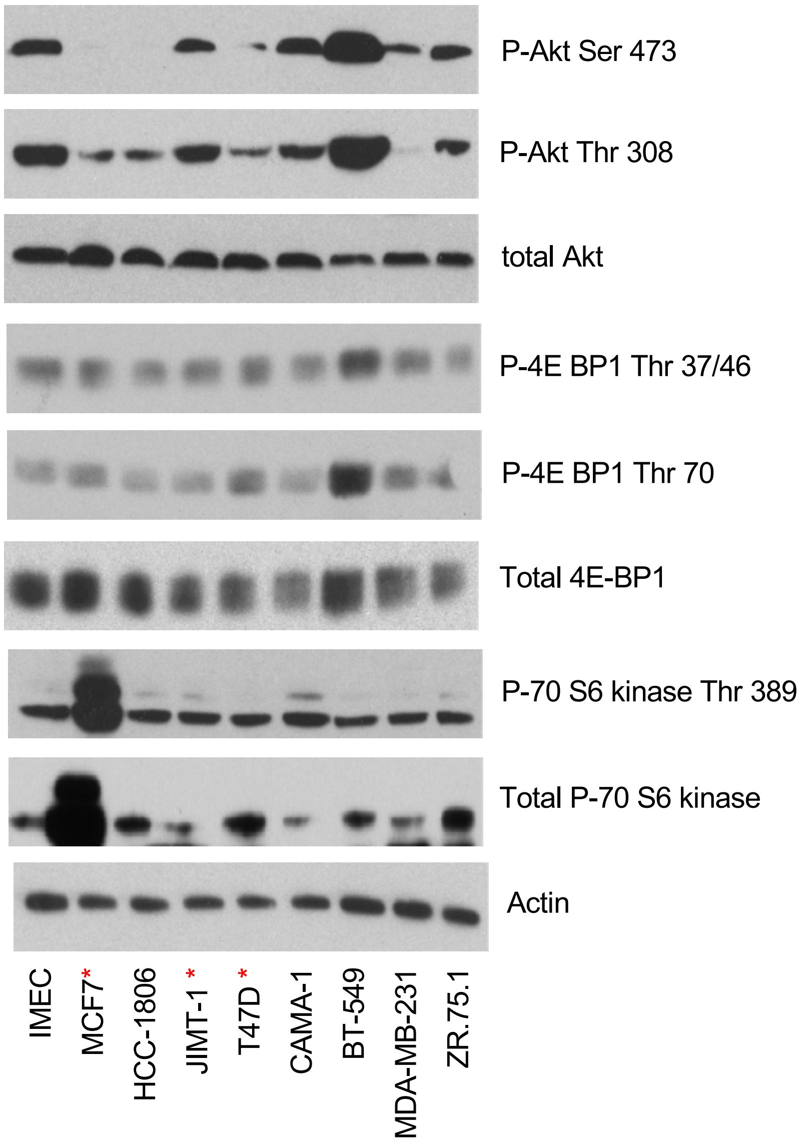

Supplemental Figure 7 Analysis of PI3K signalling in breast cancer cell lines

The cell lines indicated, under log-phase growth, were analysed for expression of the proteins and phosphor-epitopes indicated.

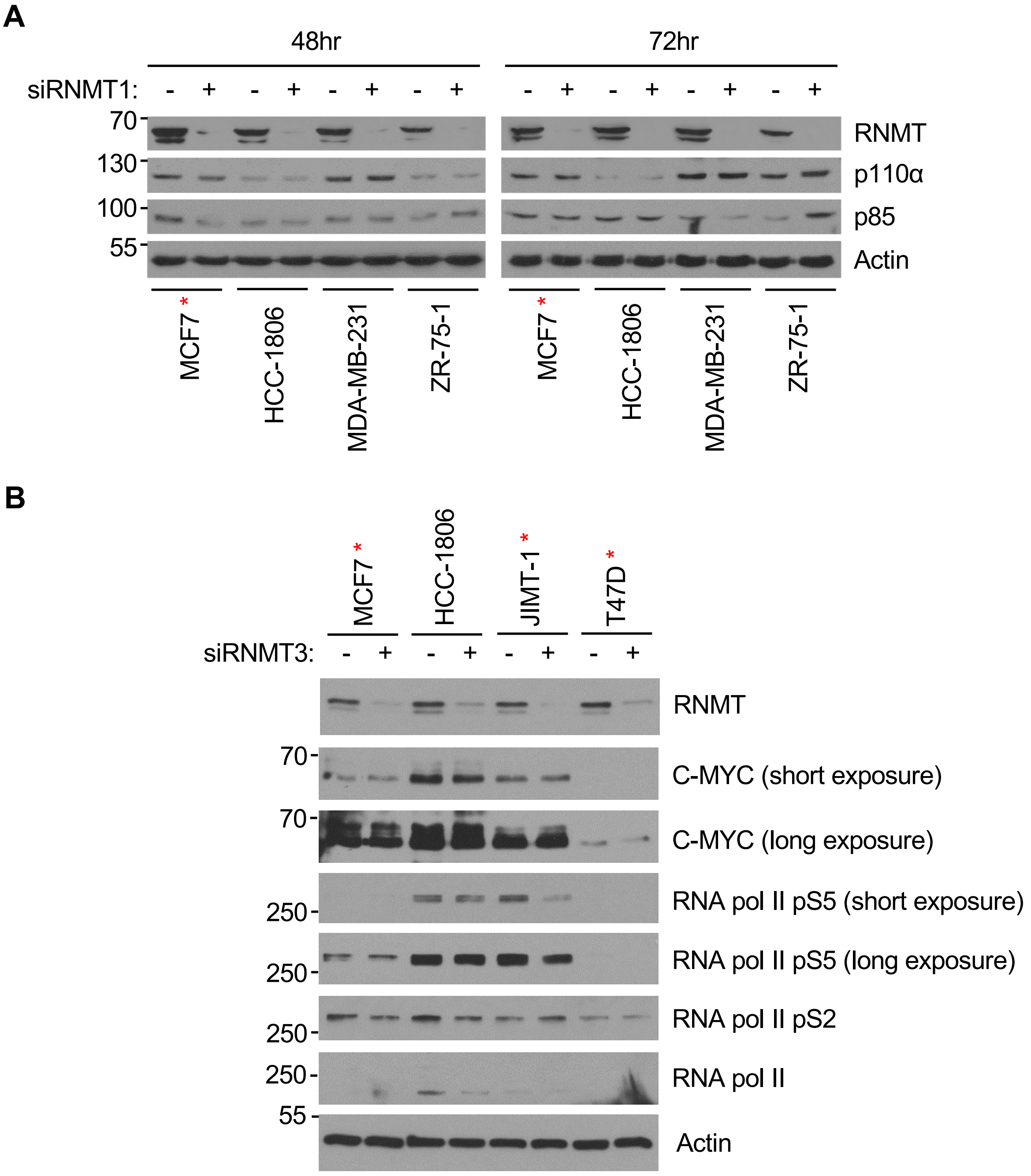

Supplemental Figure 8 Analysis of the impact of RNMT siRNA transfection on PI3Kα subunits, c-Myc and RNA pol II phosphorylation.

The cell lines indicated were transfected with RNMT siRNA for a) 48hrs and 72hrs; and b) 72 hrs. Cell extracts were analysed for expression of the proteins and phosphor-epitopes indicated.
